# The Default Mode Network and Visual Network Functional Connectivity Changes in Noise‐Induced Hearing Loss Patients: A Resting‐State fMRI Study

**DOI:** 10.1002/brb3.70465

**Published:** 2025-04-02

**Authors:** Wei Lian, Lei Zhang, Aijie Wang, Ranran Huang, Haijun Zhang, Xianghua Bao, GuoweiZ hang

**Affiliations:** ^1^ Department of Radiology Yantaishan Hospital Yantai People's Republic of China; ^2^ Department of Occupational Yantaishan Hospital Yantai People's Republic of China

**Keywords:** default mode network (DMN), functional connectivity (FC), functional magnetic resonance imaging (fMRI), noise‐induced hearing loss (NIHL), visual network (VN)

## Abstract

**Background:**

Hearing loss affects communication and hinders personal attention and cognitive ability. We hypothesized that noise‐induced hearing loss (NIHL) patients during long‐term noise exposure may result in multimodal plastic changes in the nonauditory central nervous system.

**Objective:**

To investigate the functional connectivity (FC) of the default mode network (DMN) and visual network (VN) in patients with occupational NIHL using resting‐state functional magnetic resonance imaging (rs‐fMRI).

**Methods:**

Ninety‐eight people with NIHL and 78 healthy controls (HCs) matched for age and educational level were enrolled. The mini–mental state examination (MMSE) was conducted, and rs‐fMRI scanning was performed. The data were processed and analyzed to identify FC changes between DMN, VN, and the whole brain.

**Results:**

Compared with the HCs, the NIHL group showed significantly enhanced connectivity with multiple brain regions when utilizing the DMN as seed regions of interest (ROI), with only some brain regions showing significantly decreased connectivity. When the VN was used as the seed ROI, the NIHL group showed significantly enhanced connectivity with multiple brain regions (corrected by GRF, *p *< 0.05). In the present study, the FC between multiple brain areas of VN and DMN in the NIHL patient group was enhanced compared to the normal population. The phenomenon of “perceptual compensation” is confirmed. The results of this study suggest that NIHL causes various changes in brain function related to emotion, decision‐making, social cognition, and psychopathology. It suggests that changes in brain functional networks involve complex processes involving plasticity and damage to multiple networks.

**Conclusions:**

The NIHL patients showed abnormal FC changes in both the DMN and VN, indicating widespread multimodal plasticity and reorganization of nonauditory central nervous system functions in people with NIHL.

## Graphical abstract

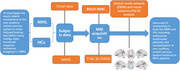

**Abbreviations**
BOLD: blood oxygenation level‐dependent
CAL: calcarine fissure
CUN: cuneus cortex
DMN: default mode network
EEG: electroencephalogram
EPI: echo‐planar imaging
FC: functional connectivity
FFG: fusiform gyrus
FNC: functional network connectivity
FOV: field of view
GRF: Gaussian random field
HCs: healthy controls
IFGtriang: Inferior frontal gyrus, triangular part
IPL: lateral parietal lobe
L: Left
MMSE: mini–mental state examination
MNI: Montreal Neurological Institute
MOG: middle occipital gyrus
MTG: middle temporal gyrus
NIHL: noise‐induced hearing loss
ORBinf: Inferior frontal gyrus, orbital part
ORBmid: middle frontal gyrus orbital region
ORBsup: Superior frontal gyrus, orbital part
PCG: posterior cingulate gyrus
PCUN: precuneus
PoCG: postcentral gyrus
R: Right
ROI: regions of interest
rs‐fMRI: resting‐state functional magnetic resonance imaging
RSNs: resting‐state networks
SNHL: sensorineural hearing loss
SOG: superior occipital gyrus
VN: visual network

## 1 Introduction

Occupational noise‐induced hearing loss (NIHL) is a gradually progressive, bilateral, and symmetric sensorineural hearing loss (SNHL), which is primarily caused by damage to the inner ear or auditory nerve resulting in auditory impairment. It occurs in individuals with prolonged occupational exposure to noise levels exceeding national health standards, regardless of the use of effective hearing protection (Lie et al. 2016). Previous studies have shown that workers in industries such as construction, manufacturing, mining, agriculture, utilities, transportation, military personnel, and musicians are at the highest risk for NIHL (Masterson et al. 2016).

Research has indicated that hearing loss affects many aspects of an individual's life, with auditory loss affecting communication and hindering personal attention and cognitive ability (Basner et al. 2014). Cognitive decline is common among individuals with hearing loss, including impairments in language development (Cupples et al. 2018) and executive function among adolescents with profound deafness (Hall et al. 2018). Elderly individuals with mild hearing loss have twice the risk of developing dementia, whereas those with severe hearing loss have five times the risk (Lin 2012). Current management strategies for NIHL primarily focus on improving auditory system symptoms, such as hearing loss and tinnitus. However, clinical behavioral studies have suggested that participants with NIHL not only suffer from auditory system‐related damage but also may exhibit psychological disorders such as anxiety and depression (Feng et al. 2022; Kuleshova and Pankov 2021).

Resting‐state functional magnetic resonance imaging (rs‐fMRI) is being increasingly used for noninvasive studies of the central nervous system due to its advantages of simplicity and high subject compliance. Compared to resting‐state networks (RSNs) utilizing electroencephalogram (EEG) data, rs‐fMRI analysis is a mainstream research technique (Xie 2022), and the default mode network (DMN) plays a central role in this work (Raichle 2015). The DMN is a crucial RSN that is activated when the brain is in a task‐free resting state and plays a vital role in self‐referential mental activity, cognitive control, and maintenance of internal and external attention (Leech and Sharp 2014). Based on structural differences in the brain, DMN encompasses four brain regions (Gusnard et al. 2001): (1) the posterior cingulate cortex area, comprising the posterior cingulate cortex, precuneus, and cuneus; (2) the lateral parietal cortex area, comprising the bilateral inferior parietal lobules, temporal lobes, and occipital lobes; (3) the medial prefrontal cortex area; and (4) the dorsomedial prefrontal cortex area. There is increasing evidence to suggest that DMN is involved in the functional modulation of SNHL, which is driven by auditory deprivation (Schmidt et al. 2013). The prior study based on independent component analysis also found that functional network connectivity (FNC) between DMN and VN in people with NIHL was all lower than in health controls (HCs) (Ranran et al. 2024). The visual network (VN) is a sensory system comprising the primary VN (medial visual cortex) and lateral VN (high‐level visual cortex), which are associated with visual attention and other behaviors. The brain regions involved in the VN (Y. L. Yang et al. 2015) include the bilateral fissure and surrounding cortex, in which the lateral occipital cortex is located in the visual area, which plays an important role in object recognition, spatial coordination, and motion perception, and has close structural connections to other cortical areas (Palejwala et al. 2020).

While studies on general hearing loss populations (e.g., adolescents with congenital deafness or elderly individuals with age‐related hearing loss) suggest broader cognitive and psychological impacts, our focus on occupational NIHL highlights similar multimodal central nervous system changes arising specifically from noise‐induced auditory deprivation. Currently, no studies have yet performed a correlation analysis between brain regions of the DMN and VN in people with NIHL as seed points with whole brain voxels separately. Based on previous research, we hypothesized that long‐term noise exposure leading to auditory deprivation may result in multimodal plastic changes in the nonauditory central nervous system. Therefore, in this study, we selected various brain regions of the DMN and VN as the regions of interest (ROI) for seed‐based whole‐brain functional connectivity (FC) analysis to further explore the multimodal plastic mechanism of the nonauditory central nervous system in NIHL. This study provides evidence to show the multimodal plastic mechanism of the nonauditory central nervous system in NIHL and will help in the early implementation of preventive interventions, such as reducing the contact time, such as taking breaks in the soundproof room, or reducing the daily and weekly contact noise time. You can also rotate the type of work according to the actual situation, measures to improve the prognosis of NIHL.

## 2 Materials and methods

### 2.1 General Information

This study enrolled 98 patients diagnosed with occupational NIHL between 2014 and 2023 in our hospital's occupational disease department. Concurrently, 78 healthy volunteers matched for age and education level were selected as the healthy controls (HCs) group. As most participants with NIHL in this region are primarily involved in works such as mining, drilling, welding, and grinding, all NIHL participants were male. HCs were also male to ensure demographic matching. All participants were examined using the mini–mental State Examination (MMSE) and the Hamilton Anxiety Scale (HAMA). Clinical data, including age, education, hearing levels, occupation, and duration of noise exposure, were also collected. All participants provided informed consent to participate.

Diagnostic Criteria for NIHL (Leech and Sharp 2014): In accordance with the guidelines outlined in the “Diagnosis of Occupational NIHL: GBZ 49–2014,” included: Based on an occupational noise operation history of more than 3 years, progressive hearing loss, tinnitus, and other symptoms, and pure tone audiometry results combined with occupational health monitoring data and on‐site occupational health investigation, comprehensive analysis, and exclusion of other causes of hearing damage. The average hearing thresholds for the binaural high frequency (3000, 4000, and 6000 Hz) (> 40 dB), better whisper frequency (500, 1000, and 2000 Hz), and high frequency (4000 Hz) were used for diagnosis classification—mild: 26–40 dB; moderate: 41–55 dB; severe: ≥ 56 dB. For this standard, “noise work” refers to work in an environment with noise intensity exceeding the “occupational exposure limit for hazardous factors in the workplace,” which is an 8‐h‐equivalent sound level (A‐weight) of > 85 dB.

Inclusion Criteria for Participants: To ensure the homogeneity and validity of the study sample, the following inclusion criteria were established. Demographic Characteristics: Adult males. Han ethnicity. Education Level: Ranging from elementary school to college. Cognitive Function Assessment: an MMSE score exceeding 27 points (indicating intact cognitive function).

Exclusion Criteria for Participants: To ensure the integrity and reliability of the results, participants who met any of the following criteria were excluded: Medical History: History of cranial surgery or neurological diseases. Hearing Impairment: Hearing impairment attributed to nonoccupational causes. Psychiatric and Substance Use History: History of alcohol or psychotropic drug abuse. Diagnosed psychiatric disorders and family history thereof. MRI contraindications: claustrophobia or presence of metal implants, or other contraindications for magnetic resonance imaging (MRI). Medication History: use of sedatives, analgesics, antidepressants, or tranquilizers within the past two weeks. Cooperation and Participation: Unable to cooperate with MR scanning. Prior Participation: Participation in other clinical trials that could influence the study results.

### 2.2 Functional MRI Data Acquisition

MRI Scanning Protocol: A GE Discovery MR 750 3.0 T scanner equipped with an eight‐channel head coil was used for imaging procedures. All participants were positioned in the supine position with eyes closed, earplugs inserted for hearing protection, and the head securely immobilized.

Structural brain imaging parameters are as follows: T1 3D‐FSPGR sequence EPI: TR: 6.9 ms, TE: 3.4 ms, slice thickness: 1 mm with no interslice gap, FOV: 25.6 cm × 25.6 cm, matrix: 256 × 256, flip angle of 12°, 178 slices. rs‐fMRI sequence as follows: TR: 2000 ms, TE: 35 ms, slice thickness: 4 mm, with no interslice gap, FOV: 24 cm × 24 cm, matrix: 64 × 64, flip angle: 90°, 200 volumes across 40 slices.

### 2.3 fMRI Data Processing

Preprocessing of the rs‐fMRI data was performed using DPARSF software in the MATLAB 2023 platform (http://rfmri.org/DPARSF), as follows:

1. Image format conversion and removal of the first 10 time points.
2. Temporal and motion correction, excluding data with head motion > 1.5 mm or head rotation > 1.5.
3. Spatial normalization of the corrected images in accordance with the Montreal Neurological Institute (MNI) standard template and voxel resampling to 3 mm × 3 mm × 3 mm.

FC analysis was performed as follows:

1. Low‐frequency temporal filtering of preprocessed data was performed using the CONN toolbox (http://www.nitrc.org/projects/conn), with a bandpass filter of 0.01–0.08 Hz to remove high‐frequency physiological noise and low‐frequency drift.
2. Regression analysis was performed to remove confounding factors, such as the white matter, cerebrospinal fluid, and motion parameters.

Extraction of seed points for differential brain regions was performed utilizing the AAL116 template (Tzourio‐Mazoyer et al. 2002), selecting the DMN (including the bilateral precuneus [PUCN.BIL], left/right inferior parietal lobule [IPL.L/R], bilateral middle frontal gyrus orbital region [ORBmid.BIL]) and VN (including the left/right lateral occipital lobe [LOL.L/R], bilateral medial occipital lobes [MOL.BIL], and bilateral posterior occipital lobes [POL.BIL]) as the regions of interest for whole‐brain resting‐state FC analysis to obtain whole‐brain FC maps. The average time series of different brain regions was correlated with the time series of other voxels in the brain, while the correlation coefficient (*r*) was transformed into Fisher's *Z*‐scores to obtain FC values. Intergroup analysis was conducted with Gaussian random field (GRF) correction, cluster‐level significance set at *p* < 0.05, and voxel‐level significance set at *p* < 0.001 using bilateral tests.

### 2.4 Statistical Analysis

Data were analyzed using SPSS 27.0. Independent samples *t*‐tests were used for intergroup comparisons of all measured parameters, with statistical significance set at *p* < 0.05. Clinical data, including age, years of education, and MMSE scores, were compared between the two groups.

## 3 Results

### 3.1 Clinical Basic Information

**FIGURE 1:**
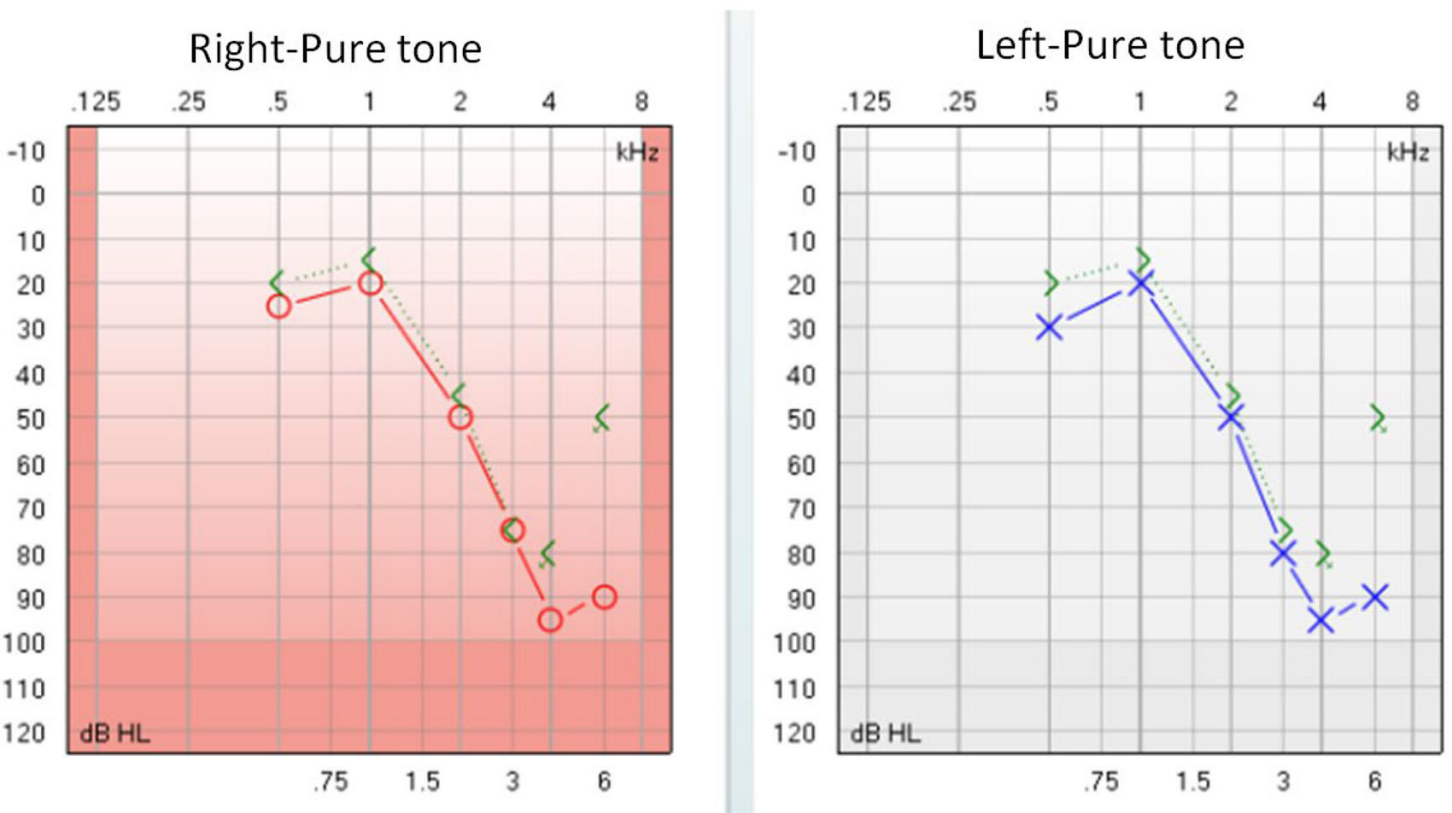
A typical audiogram of a patient (a rock driller) with NIHL. Male; age: 50 years; rock drilling; noise exposure: 8 years. Binaural symmetry sensorineural hearing loss, mainly high‐frequency hearing loss, 4 kHz as the average binaural high‐frequency hearing threshold (BHFTA) ≥ 40 dB, good auditory threshold weight > 25 dB; NIHL: noise‐induced hearing loss.

Comparisons of age (45.71 ± 6.98 years, 47.50 ± 7.64 years), years of education (10.54 ± 2.04 years, 10.68 ± 3.02 years), and MMSE scores (28.91 ± 1.33, 28.91 ± 1.15) between the NIHL and HC groups showed no statistically significant differences (*p* > 0.05) (Table 1). A typical audiogram of a patient (a rock driller) with NIHL is shown in Figure 1.

**TABLE 1:** Comparison of general clinical data between the NIHL group and the HC group (mean ± standard deviation).

Group	NIHL	HCs	*t*	*p*
Age (years)	45.71 ± 6.98	47.50 ± 7.64	−1.628	0.105
Education level (years)	10.54 ± 2.04	10.68 ± 3.02	−0.350	0.727
MMSE (score)	28.91 ± 1.33	28.91 ± 1.15	−0.001	0.999
HAMA (score)	9.09 ± 8.36	3.81 ± 1.02	−5.534	< 0.01
Noise exposure (years)	15.81 ± 8.16	—	—	—
Mean hearing threshold (dB)	42.46 ± 14.45	—	—	—
Occupation (%)	Rock drilling (42%) Weld (13%) Polish (12%) Other (33%)	—	—	—

*Note*: Noise exposure and time: This refers to the long‐term occupational exposure to noise levels exceeding the national health standards, typically in environments where the 8‐hour equivalent sound level (A weight) is greater than 85 dB; time: This detail refers to the duration for which the patient has worked in a noisy work environment. It is measured in years and indicates the length of time the patient has been exposed to occupational noise. A two independent samples *t*‐test was used, and *p *< 0.05 was statistically different. Mean hearing threshold: The average hearing thresholds for the binaural high frequency (3000, 4000, and 6000 Hz) (> 40 dB), better whisper frequency (500, 1000, and 2000 Hz), and high frequency (4000 Hz) (> 40 dB) were used for diagnosis and diagnostic classification—mild: 26–40 dB; moderate: 41–55 dB; severe: ≥ 56 dB. A two independent samples *t*‐test was used, and *p *< 0.05 was statistically different.

Abbreviations: HCs: health controls; NIHL: noise‐induced hearing loss.

### 3.2 DMN and VN FC Analysis

#### 3.2.1 DMN Brain Region vs. Whole‐Brain FC Analysis

**FIGURE 2:**
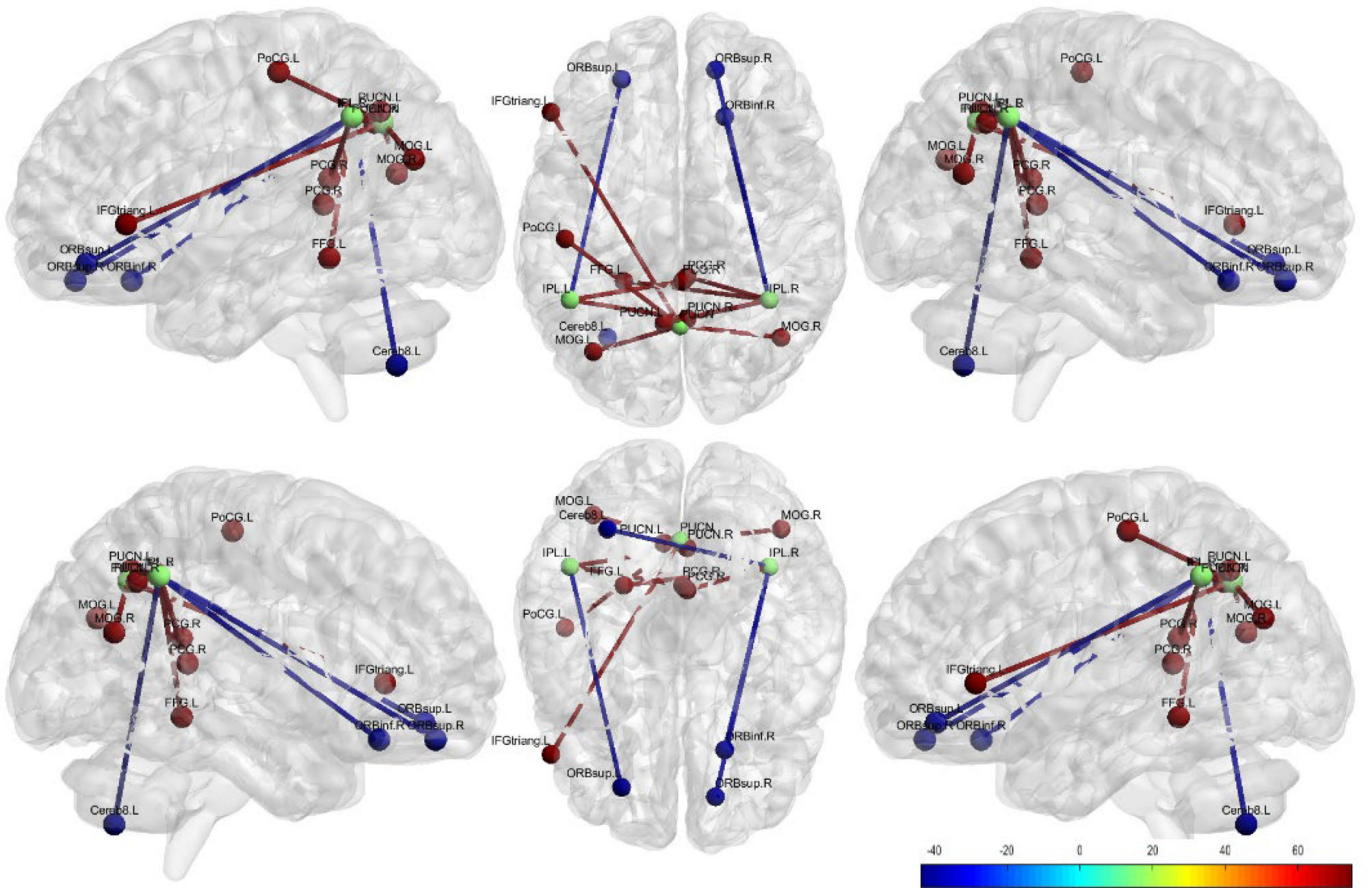
Abnormal FCs between various sub‐ROIs of DMN and whole‐brain ROI in the NIHL group versus the HC group. DMN: default mode network; FC: functional connectivity; HCs: health controls; NIHL: noise‐induced hearing loss. Each node represents a brain region with significant differences in FC. Green spheres represent seed points of the DMN ROI, red spheres and lines represent brain regions with increased FC, and blue spheres and lines represent brain regions with decreased FC; The color scale represents the connection strength; bright color represents the increase, and dark color represents the decrease.

The results of the DMN brain region versus whole‐brain FC analysis are shown in Table 2 and Figure 2.

**TABLE 2:** Abnormal FCs between various sub‐ROIs of DMN and whole‐brain ROI in the NIHL group versus the HC group.

DMN ROI (peak MNI coordinates)	Abnormal whole‐brain ROI	Peak MNI coordinates			Voxels	FC in NIHL vs. HCs	*t*
		*X*	*Y*	*Z*			
Bilateral precuneus (1, −61, 38)	Middle occipital gyrus_L	−36	−72	30	504	Increased	4.96
	Middle occipital gyrus_R	44	−66	25	294	Increased	4.95
	Postcentral gyrus_L	−48	−24	56	180	Increased	4.95
	Inferior frontal gyrus, triangular part_L	−54	30	2	181	Increased	5.11
Left lateral parietal lobe (−46, −50, 40)	Precuneus_R	4	−58	38	947	Increased	5.35
	Posterior cingulate gyrus_R	2	−42	18	340	Increased	5.35
	Superior frontal gyrus, orbital part_L	−24	44	−12	69	Decreased	−5.27
right lateral parietal lobe (39, −50, 40)	Precuneus_L	−06	−60	42	542	Increased	4.73
	Fusiform gyrus_L	−23	−42	−10	54	Increased	4.55
	Posterior cingulate gyrus_R	4	−40	09	89	Increased	5.08
	cerebellum 8_L	−30	−66	−48	72	Decreased	−3.93
	Inferiorfrontal gyrus, orbital part_R	20	28	−18	115	Decreased	−4.17
	Superior frontal gyrus, orbital part_R	16	48	−18	74	Decreased	−4.17

*Note*: Intergroup analysis was conducted with GRF correction, cluster‐level significance set at *p* < 0.05, and voxel‐level significance set at *p* < 0.001 using bilateral tests.

Abbreviations:

DMN, default mode network; FC, functional connectivity; HCs, health controls; MNI, Montreal Neurological Institute; NIHL, noise‐induced hearing loss.

The PUCN.BIL as the seed ROI: Compared with the HCs group, the increased FC was noted between PUCN.BIL and the following regions in the NIHL group: left middle occipital gyrus (MOG.L), *t* = 4.96, *p* < 0.05; right middle occipital gyrus (MOG.R), *t* = 4.95, *p *< 0.05; Left Postcentral Gyrus (PCG.L), *t* = 4.95, *p* < 0.05; and Left Inferior Frontal Gyrus Triangular Part (IFGtriang.L), *t* = 5.11, *p *< 0.05.

The IPL.L as the seed ROI: Compared with the HCs group, the significant alterations in FC were noted in the NIHL group. Increased FC was noted between IPL.L and the Right Precuneus (PUCN.R): *t* = 5.35, *p* < 0.05 and Right Postcentral Gyrus (PCG.R): *t* = 5.35, *p* < 0.05. Decreased FC was observed between the IPL.L and the left superior frontal gyrus (ORBsup.L): *t* = −5.27, *p* < 0.05.

The IPL.R as the seed ROI: Compared with the HCs group, the significant alterations FC was noted in the NIHL group. Increased FC was noted between IPL.R and the Left Precuneus (PUCN.L): *t* = 4.73, *p *< 0.05; Left Fusiform Gyrus (FFG.L): *t* = 4.55, *p* < 0.05; and PCG.R: *t* = 5.08, *p* < 0.05. Decreased FC was observed between IPL.R and the following regions: Left Cerebellum 8 (Cereb8.L): *t* = −3.93, *p* < 0.05; Right Inferior Frontal Gyrus (ORBinf.R): *t* = −4.17, *p* < 0.05; Right Superior Frontal Gyrus (ORBsup.R): *t* = −4.17, *p* < 0.05.

The ORBmid.BIL as the seed ROI: compared with the HCs group, no significant alterations in FC were noted in the NIHL group (*p* > 0.05).

#### 3.2.2 VN Brain Region vs. Whole‐Brain FC Analysis

**FIGURE 3:**
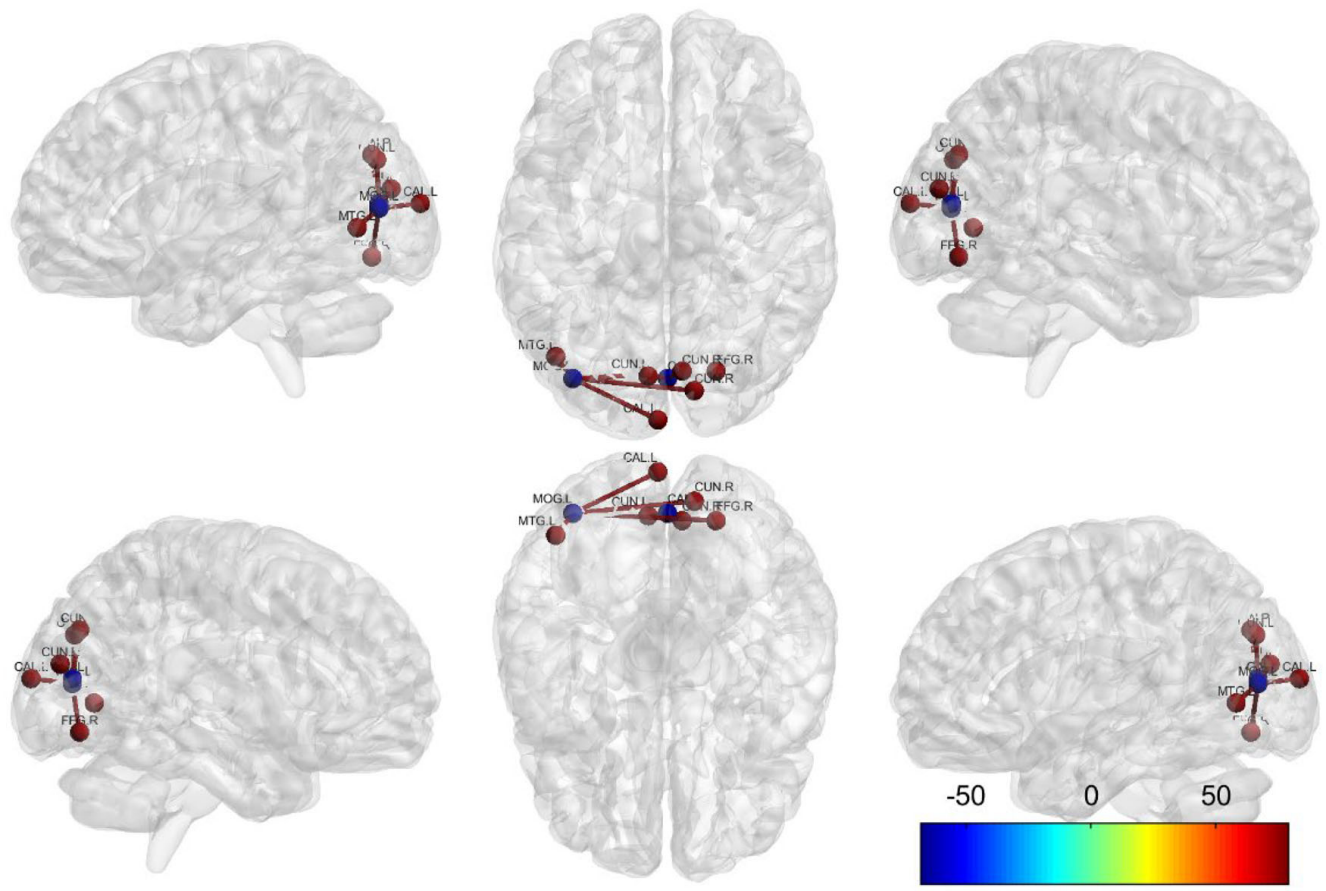
Abnormal FCs between various sub‐ROIs of VN and whole‐b rain ROI in the NIHL group versus the HC group. VN: visual network; FC: functional connectivity; HCs: health controls; NIHL: noise‐induced hearing loss. Each node represents a brain region with significant differences in FC. Blue spheres represent the seed points of various VN ROIs, whereas the red spheres and red lines represent brain regions with enhanced FC; the color scale represents the connection strength, bright color represents the increase, and dark color represents the decrease.

The results of the VN Brain Region versus Whole‐Brain FC Analysis are shown in Table 3 and Figure 3.

**TABLE 3:** Abnormal FCs between various sub‐ROIs of VN and whole‐Brain ROI in the NIHL group versus the HC group.

		Peak MNI coordinates					
VN ROI (Peak MNI coordinates)	Abnormal FC whole‐brain ROI	*X*	*Y*	*Z*	Voxels	FC in NIHL vs. HCs	t
left lateral occipital lobe (−37,−79,10)	Cuneus_R	28	13	−84	28	Increased	5.02
	Fusiform gyrus_R	84	22	−76	84	Increased	5.04
	Calcarine_L	154	−2	−96	154	Increased	5.63
	Middle temporal gyrus_L	369	−44	−70	369	Increased	5.22
bilateral medial occipital lobes (2,−79,12)	Cuneus_L	95	−6	−78	95	Increased	4.42
	Cuneus_R	40	8	−76	40	Increased	4.42

*Note*: Intergroup analysis was conducted with GRF correction, cluster‐level significance set at *p *< 0.05, and voxel‐level significance set at *p *< 0.001 using bilateral tests.

Abbreviations: FC, functional connectivity; HCs, health controls; MNI, Montreal Neurological Institute; NIHL, noise‐induced hearing loss; VN, visual network.

The LOL.L as the seed ROI: Compared with the HCs group, the increased FC was noted between LOL.L and the following regions in NIHL group: right cuneus (CUN.R): *t* = 5.02, *p* < 0.05; right fusiform gyrus (FFG.R): *t* = 5.04, *p* < 0.05; left calcarine (CAL.L): *t* = 5.63, *p* < 0.05; left middle temporal gyrus (MTG.L): *t* = 5.22, *p *< 0.05.

The MOL.BIL as the seed ROI: Compared with the HCs group, the increased FC was noted between the MOL.BIL and the Left Cuneus (CUN.L): *t* = 4.42, *p* < 0.05 and CUN.R: *t* = 4.42, *p* < 0.05.

The LOL.R and POL.BIL as the seed ROI: compared with the HCs group, no significant alterations in FC were noted in the NIHL group (*p* > 0.05).

## 4 Discussion

The commonly used diagnostic assessment methods for NIHL include pure tone audiometry, tympanometry, auditory brainstem response testing, and otoacoustic emission testing. However, these techniques cannot assess changes in the central nervous system related to noise‐induced deafness. In recent years, fMRI has become a valuable tool for the noninvasive exploration of structural and functional abnormalities in the brain, which can detect brain damage before the development of behavioral abnormalities. This technique utilizes blood oxygenation level‐dependent (BOLD) signal changes in the brain tissue to indirectly reflect neuronal activity. Studies on specific populations can provide insight into the mechanisms of functional reorganization and guide potential interventions.

In this study, compared to HCs, participants with NIHL exhibited abnormal FC between the DMN and various seed points in the VN and the entire brain. Specifically, in the DMN regions, enhanced FC was observed between the PUCN. BIL and the MOG.L, MOG.R, PoCG.L, and IFGtriang.L. Enhanced FC was also observed between the IPL.L, PUCN.R, and PCG.R, while a decreased FC was observed with the ORBsup.L. Moreover, enhanced FC was noted between the right IPL.R and PUCN.L, FFG.L, and PCG.R, with decreased FC in the Cereb8.L, ORBinf.R, and ORBsup.R. In the VN regions, enhanced FC was observed between the LOL.L and the CUN.R, FFG.R, CAL.L, and MTG.L. Additionally, enhanced FC was observed between the MOL.BIL, CUN.L, and CUN.R. These abnormalities in seed‐based FC suggest that changes in brain function in participants with NIHL involve a wide range of nonauditory central brain areas, including multiple functional networks that are multimodal and plastic, leading to reorganization.

### 4.1 DMN Brain Region vs. Whole‐Brain FC Analysis

DMN involves brain regions that primarily include the medial prefrontal cortex, posterior cingulate cortex, precuneus, temporal lobes, anterior cingulate cortex, inferior parietal lobule, and hippocampus (Raichle 2015). In this study, the DMN‐related abnormal FC brain regions mainly included the PUCN. BIL, IPL. BIL. The PUCN is an important part of the DMN, an important structure of FC within the brain, and an important part of obtaining information from the outside world and integrating information obtained from different regions (Buckner et al. 2008). The function of PUCN is divided into four main categories: visuospatial imagery, episodic memory retrieval, self‐processing, and consciousness (Cavanna and Trimble 2006). The brain regions with increased FC with PUCN were mainly concentrated in MOG, PCG, and IFG. The MOG showed a preference for spatial over nonspatial processing of both auditory and tactile stimuli. Furthermore, MOG activity was correlated with the accuracy of individual sound localization performance (Renier et al. 2010). The PCG is in the parietal lobe and serves as the central sensory area, playing a core role in processing sensory information from various parts of the body, sensorimotor integration, pain stimulus processing, and regulation of negative emotions and fear memory (L. Yang et al. 2022). The IFG, located in the medial frontal lobe, is a key cortical center in the emotion and cognitive control circuit and is predominantly involved in decision‐making (Hayakawa et al. 2012, Roberts et al. 2016). Based on these changes, we hypothesized that due to the hearing loss in the NIHL group, the abnormal FC of MOGs suggests increased compensatory activity, which contributes to the accuracy of individual sound localization. Since the HAMA of the NIHL group was significantly higher than that of the HC group, the abnormal FC of PCG and IFG may be related to the enhancement of negative emotion regulation and cognitive control.

The IPL has a wide range of functions and is primarily responsible for sensory integration, language integration, attention functions, writing, and spatial localization (Sijia and Hua 2019). The brain regions with increased FC with IPL were mainly concentrated in PUCN, PCG, and FFG, and decreased in ORBsup ORBinf, and Cereb8.L. The highest level of cortical visual processing occurs in the temporal lobe, with the FFG representing the largest component of the ventral temporal lobe in humans The FFG is considered to be a key structure for professional computation of advanced visual functions, such as face perception, object recognition, and reading (Weiner and Zilles 2016). The CUN, a part of the DMN, is involved in multisensory integration and cognitive processing (Tomasi and Volkow 2011). Therefore, we hypothesize that the enhancement of PUCN, PCG, and FFG in people with NIHL is a compensatory change in the brain in response to hearing loss, including enhanced higher visual functions, cognitive processing, and regulation of negative emotions. ORBsup, and ORBinf mainly belong to the frontal lobe and are predominantly involved in the regulation of negative emotions and traumatic memory (L. Yang et al. 2022), as well as in the processing of social and emotional information and complex cognitive functions (Wendelken and Bunge 2010). Normal frontal lobe function can help to improve fear memory and emotions. Therefore we speculate that the anxiety changes in NIHL may be related to the abnormal disconnection between brain networks. The cerebellum is essential for maintaining balance and regulating movement. Recent research has indicated that the cerebellum is also involved in other processes, including auditory conduction and processing (Boyen et al. 2014 ). Therefore, we speculate that the changes in anxiety in people with NIHL may be related to the abnormal disconnection of brain networks in ORBsup, ORBinf, and other regions, and the abnormal connection with the cerebellum also suggests that hearing loss may be related to the reduction of auditory conduction and auditory processing in the cerebellum。

Combined with HAMA, we speculated that the abnormalities in the FC of DMN nodes suggest disturbances in various sensory integration functions in participants with NIHL, due to long‐term exposure to noisy environments, which may be related to anxiety and negative emotional disorders while mirroring the adaptive functional reconfiguration of brain performance. Furthermore, the study by Agnès Job (Job et al. 2004) provides additional support for the link between emotional states and auditory function. They found that higher levels of tension‐anxiety were associated with a greater risk of tinnitus onset and significant decreases in DPOAEs at 3 kHz after exposure to impulse noise. This suggests that the fMRI changes observed in people with NIHL, which indicate increased anxiety, could be related to actual clinical outcomes such as tinnitus and changes in cochlear sensitivity. All these findings were partially consistent with previous clinical studies evaluating NIHL (Ranran et al. 2024), confirming that NIHL‐related mood alterations are complex regulatory processes involving the adjustment and coordination of higher‐order networks. Moreover, in the study of Zhang et al. (2014), it was found that long‐term unilateral SNHL contributes to changes in the DMN, which might affect cognitive abilities; these changes predate behavioral measurements, suggesting that the FC might be more sensitive for observing cognitive changes in patients with hearing loss than clinical neuropsychological tests.

### 4.2 VN Brain Region vs. Whole‐Brain FC Analysis

The primary visual cortex predominantly includes the calcarine sulcus, cuneus, lingual gyrus, and other brain areas, whereas the secondary visual cortex mainly includes the superior, middle, and inferior occipital gyri, as well as the adjacent temporo‐occipital areas (Wandell et al. 2007). In this study, the VN‐related abnormal FC brain regions mainly included the LOL, MOL.BIL. LOL, MOL.BIL belong to the occipital cortex, which is situated in the visual area of the brain, which forms part of the VN, where it plays an important role in object recognition, visual–spatial coordination, and motion perception, and is closely connected with other cortical areas (Palejwala et al. 2020). Visual processing is believed to begin in the occipital lobe cortex and progress through the ventral and dorsal pathways. The brain regions with increased FC with LOL were mainly concentrated in CUN, FFG, CAL, and MTG, and the brain regions with increased FC with MOL.BIL were mainly concentrated in CUN. The MTG is understood to play a role in language‐related tasks such as lexical comprehension and semantic cognition (Briggs et al. 2021). In this study, both LOL.L and MOL.BIL had increased FC with CUN, suggesting that long‐term hearing deprivation and FC changes between different networks after loss are closely related to hearing impairment.

Yanping et al. (2002) used fMRI to investigate the excitability of the occipital lobe visual cortex in deaf individuals stimulated with fixed‐frequency, varying‐intensity point light sources, which indicated that when deaf and healthy individuals were exposed to light stimulation, the activity of neurons in the visual cortices increased. When the intensity of external light stimulation was increased, the activation range of the visual cortex in deaf patients was found to be significantly larger than that in normal individuals, further suggesting a “perceptual compensation” effect of the sensory system in deaf individuals caused by hearing loss. In this study, the increased FC in the VN‐related ROI might again verify that long‐term hearing loss can lead to enhanced compensatory function in VN regions. In another study, Liu et al. (Liu and Liu 2021) demonstrated that bilateral congenital SNHL in children resulted in reduced ReHo in auditory and language‐related brain areas, as well as enhanced visual and spatial localization abilities, indicating that early auditory deprivation leads to decreased auditory and language processing abilities and increased visual functional compensation.

In the present study, the FC between multiple brain areas of VN and DMN in the NIHL patient group was enhanced compared to the normal population, once again confirming the phenomenon of “perceptual compensation,” which is consistent with the results of Ranran et al. (2024), who performed independent component RSN studies. Through research results, we speculate that long‐term noise exposure‐induced hearing loss not only leads to functional reorganization within the DMN but also results in perceptual compensation phenomena in external brain regions such as the VN. This hypothesis is consistent with the findings of Xing et al. (2020). The enhanced FC connections identified in these nodes reflect an increase in the allocation of neural resources for visual motion or spatial localization to compensate for weakened auditory signals after auditory deprivation.

### 4.3 Limitations

Owing to occupational constraints, we did not enroll any female patients; therefore, all participants were male, which is a major limitation of this study. Therefore, the applicability of these results to female patients is limited. In addition, the selection of only two networks, DMN and VN, is a limitation to evaluate changes in the FC of other brain networks. Another limitation is that this study did not evaluate the emotional and sleep states of patients. Additionally, it would be valuable to explore the correlation between the time since the onset of hearing loss and the observed connectivity changes. While this study did not directly assess this relationship, future research could investigate whether there is a correlation between the duration of NIHL and the strength of the observed connectivity changes. For example, patients who have lived longer with NIHL may have developed coping strategies for novel environments, which could influence the connectivity patterns observed in fMRI. Furthermore, the impact of practice and experience with fMRI procedures on the detected connectivity changes should be considered. Patients who are more familiar with the fMRI environment may exhibit different connectivity patterns compared to those who are less experienced. A short discussion on the role of experience and practice in fMRI outcomes could provide additional insights into the observed changes. A follow‐up data analysis will be conducted to increase the reliability and consistency of the study. Finally, follow‐up data on the changes after drug treatment or removal from noisy environments are lacking. Longitudinal study results would be more meaningful if people with NIHL were systematically observed and studied continuously over a longer period of time to obtain dynamic changes in the FC of the brain in the disease. These limitations should be addressed in future studies with continuous monitoring of patients.

## 5 Conclusions

This study revealed the differences in functional connections within and between the DMN and VN‐related brain areas in participants with NIHL, with enhanced or weakened connectivity in varying areas. This suggests that changes in brain function in participants with NIHL involve a wide range of nonauditory central nervous system multimodal plasticity and reorganization, indicating that changes in brain functional networks involve complex processes involving plasticity and damage across multiple networks. The results of this study indicated that early exposure to NIHL leads to various changes in brain functions related to emotions, decision‐making, social cognition, and psychopathology, providing evidence to facilitate the early diagnosis of NIHL‐related brain damage and help implement early preventive interventions, such as reducing the contact time, such as taking breaks in the soundproof room or reducing the daily and weekly contact noise time. You can also rotate the type of work according to the actual situation to improve the prognosis of NIHL.

## Author Contributions

**Wei Lian**: data curation, investigation; methodology, writing – original draft. **Lei Zhang**: data curation, writing – review and editing. **Aijie Wang**: data curation. **Ranran Huang**: data curation. **Haijun Zhang**: data curation. **Xianghua Bao**: data curation. **Guowei Zhang**: formal analysis; supervision.

## Conflicts of Interest

The authors declare no conflicts of Interest.

## Ethics Statement

The study was conducted in accordance with the Declaration of Helsinki, and the protocol was approved by the Ethics Committee of Yantai Shan Hospital (project identification code: 2024083).

## Consent

All subjects gave their informed consent for inclusion before they participated in the study.

### Peer Review

The peer review history for this article is available at https://publons.com/publon/10.1002/brb3.70465.

## Data Availability

The data that support the findings of this study are available on request from the corresponding author. The data are not publicly available due to privacy or ethical restrictions.
